# A Simple Ventilator Designed To Be Used in Shortage Crises: Construction and Verification Testing

**DOI:** 10.2196/26047

**Published:** 2021-08-05

**Authors:** Daniel S Akerib, Andrew Ames, Martin Breidenbach, Michael Bressack, Pieter A Breur, Eric Charles, David M Gaba, Ryan Herbst, Christina M Ignarra, Steffen Luitz, Eric H Miller, Brian Mong, Tom A Shutt, Matthias Wittgen

**Affiliations:** 1 SLAC National Accelerator Laboratory Palo Alto, CA United States; 2 School of Medicine Stanford University Palo Alto, CA United States; 3 Veterans Affairs Palo Alto Health Care System Palo Alto, CA United States

**Keywords:** intensive care, critical care, medicine, ventilator, COVID-19, shortage, equipment, medical device, performance, short-term, engineering, cost, ICU, intensive care unit

## Abstract

**Background:**

The COVID-19 pandemic has demonstrated the possibility of severe ventilator shortages in the near future.

**Objective:**

We aimed to develop an acute shortage ventilator.

**Methods:**

The ventilator was designed to mechanically compress a self-inflating bag resuscitator, using a modified ventilator patient circuit, which is controlled by a microcontroller and an optional laptop. It was designed to operate in both volume-controlled mode and pressure-controlled assist modes. We tested the ventilator in 4 modes using an artificial lung while measuring the volume, flow, and pressure delivered over time by the ventilator.

**Results:**

The ventilator was successful in reaching the desired tidal volume and respiratory rates specified in national emergency use resuscitator system guidelines. The ventilator responded to simulated spontaneous breathing.

**Conclusions:**

The key design goals were achieved. We developed a simple device with high performance for short-term use, made primarily from common hospital parts and generally available nonmedical components to avoid any compatibility or safety issues with the patient, and at low cost, with a unit cost per ventilator is less than $400 US excluding the patient circuit parts, that can be easily manufactured.

## Introduction

### Background

It had been estimated that, in the United States alone, several hundred thousand to as many as 1 million ventilators would be needed to care for patients with COVID-19 [1]. At the start of the pandemic, it was estimated that the number of ventilators in the United States was between 60,000 and 160,000 [2]. Many countries and regions worldwide had even bigger gaps between needed and available ventilators to fill in a short amount of time. Urgent calls were made to increase and diversify the production of ventilators [3]. One way to fill in the gap is to design, build, and test a device (acute-shortage ventilator; ASV) that can deliver a subset of requirements that a standard intensive care unit ventilator fulfills, and many have done so [4]. Previous literature has described mechanical ventilators [5-7] and their applications [8,9]. Because the ventilator consists of common hospital parts and generally available nonmedical components, hospitals around the world will be able to use these even in situations where there are breaks in the supply chain or increasing cost of specialized components triggered by a pandemic.

### System Requirements

#### Applicable Standards

The Association for the Advancement of Medical Instrumentation Emergency Use Resuscitator Systems Design Guidance (AAMI CR503:2020 [10]) specifies modifications for critical care ventilators (ISO 80601-2-12 [11]) and general standards for electrical medical equipment (IEC 60601-1 [12]) that are applicable to simplified ventilator designs that consist of mechanical systems to squeeze self-inflating bag resuscitators.

#### Fundamental Requirements

Ventilator support needs of a COVID-19 patient can include (1) bi-level positive airway pressure for patients breathing spontaneously (AAMI CR503:2020 [10], line 43); (2) pressure-control mandatory breathing (nonspontaneous) controlled by ventilator (line 44); (3) volume-control mandatory breathing controlled by ventilator (line 45); and (4) increased inspired oxygen concentration (fraction of inspired oxygen >21%) (line 46).

AAMI CR503:2020 also summarizes sets of parameters that must be controlled and monitored to be able to properly manage the treatment a COVID-19 patient (Table 1). Alarm conditions are also specified (Table 2).

**Table 1:** Summary of requirements on key control and monitoring parameters.

Parameter	Range	Tolerance	Alarms^a^	Reference^b^
Inspiratory-to-expiratory ratio	1:1-1:4	—^c^	—	Line 56
Respiratory rate (brpm)	10-30	±2	—	Line 57
Tidal volume (mL)	350-450^d^ (250-600)^e^	±4%^f^ (± 15%)^f^	±20%	Line 59
Inspiratory pressure (cmH_2_O^g^)	<40	±4%	High, low, continuous	Line 256
FiO_2_^h^ (%)	External control^i^	External control	External control	External control

^a^Alarm conditions for these parameters are generated in compliance with ISO 80601-2-12 [11], clause 12.

^b^AAMI CR503:2020 [10] line number.

^c^Not specified.

^d^The required tidal volume range.

^e^The recommended tidal volume range.

^f^The tolerance for tidal volume is reported as bias linearity.

^g^1 cmH_2_O is equivalent to 98.07 Pa.

^h^FiO_2_: fraction of inspired oxygen.

^i^Control of fraction of inspired oxygen is established by connection to an external oxygen supply.

**Table 2:** Summary of alarm conditions related to key control parameters.

Condition	User settable	Reference	
		AAMI CR503:2020 [10]	ISO 80601-2 [11]
Low airway pressure	Yes	Line 267	12.4.101.2
High airway pressure	Yes (mechanical backup^a^)	Line 289	12.4.101.3
Continuing airway pressure	Yes	Line 312	12.4.102
Tidal volume	No	Line 321	12.4.103

^a^The high airway pressure alarm requirement also specifies that there must be a mechanical backup that prevents the airway pressure from exceeding 60 cmH_2_O (1 cmH_2_O is equivalent to 98.07 Pa).

#### General Requirements for Electrical Medical Systems

AAMI CR503:2020 [10] also specifies a set of general safety guidelines, most of which are derived from general specification for safety of electrical medical devices (IEC 60601-1 [12], in particular, clauses 5-17) that cover a number of items including labeling, electrical and mechanical safety, and documentation. Although certifying that a device meets IEC 60601-1 requirements is a complicated process, in the case of the ASV, this process will be greatly simplified for a number of reasons: (1) AAMI CR503:2020 specifically excludes some requirements and clarifies relatively simple ways in which certain requirements may be met: for example, the requirements for a backup system in the case of power failure may be met with the use of an external uninterruptible power supply. (2) The device should be electrically isolated from the patient by the entire patient breathing circuit. (3) Internally, the device should use only 12 V power, thus mitigating many potential electrical hazards. (4) The electronics should all be enclosed.

## Methods

### System Description

#### Overview

**Figure 1:**
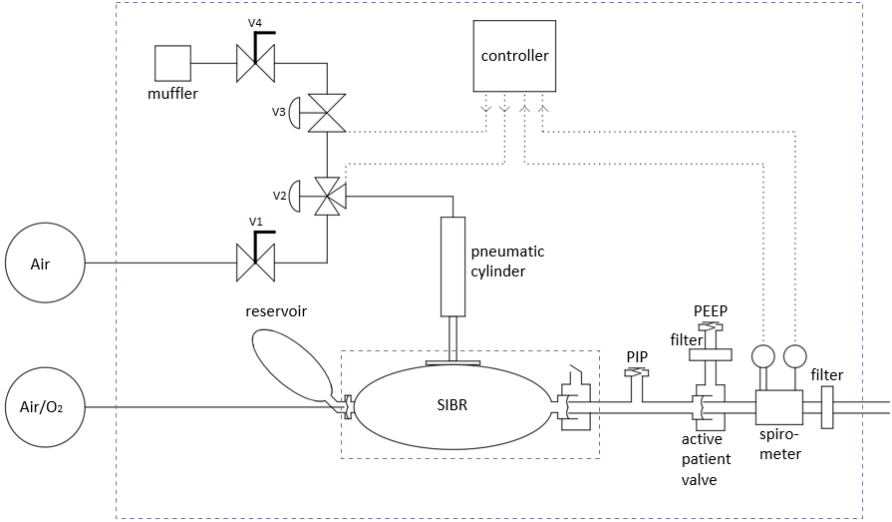
System diagram. The patient circuit, to the right, consists of standard intensive care unit parts, except for the modified PEEP valve, pressure and flow sensors, and 2 adapters. PEEP: positive end expiratory pressure; PIP: peak inspiration pressure; SIBR: self-inflating bag resuscitator.

The ASV (Figure 1) consists of the patient circuit, the mechanical and pneumatic system that compresses a self-inflating bag, sensors (spirometer), electronics, firmware, and software. The intellectual property of the ASV will be held by Stanford University and has been made available to vendors and manufacturers via open source [13].

#### Patient Circuit

The ASV patient circuit is built primarily from standard hospital components. It operates in a volume- or pressure-limited assist control mode. The patient air supply can be ambient room air or an externally supplied mixture of air and oxygen. A self-inflating bag is compressed for each inhalation cycle and is capable of delivering up to 700-800 mL of the air–oxygen mixture. The bag output is pressure-limited by the mechanical peak inspiration pressure (PIP) valve. A hose connects the PIP valve tee to the remaining components near the patient. An active patient valve serves as a check valve to minimize dead space and directs the exhalation air stream to a separate port, where a spirometer measures flow and pressure, and then passes through a heat and moisture exchanger and filter, and finally, connects to the endotracheal tube. A PEEP valve is added to the exhaust port of the active patient valve, ideally with a HEPA filter to minimize contagion. Positive end-expiratory pressure (PEEP) is a setting that ensures that the pressure at the end of either a mechanical or spontaneous breath remains above ambient. This is done to keep the small air sacs (alveoli) open when they are prone to collapse, which can occur in different lung conditions, such as acute respiratory distress syndrome.

**Figure 2:**
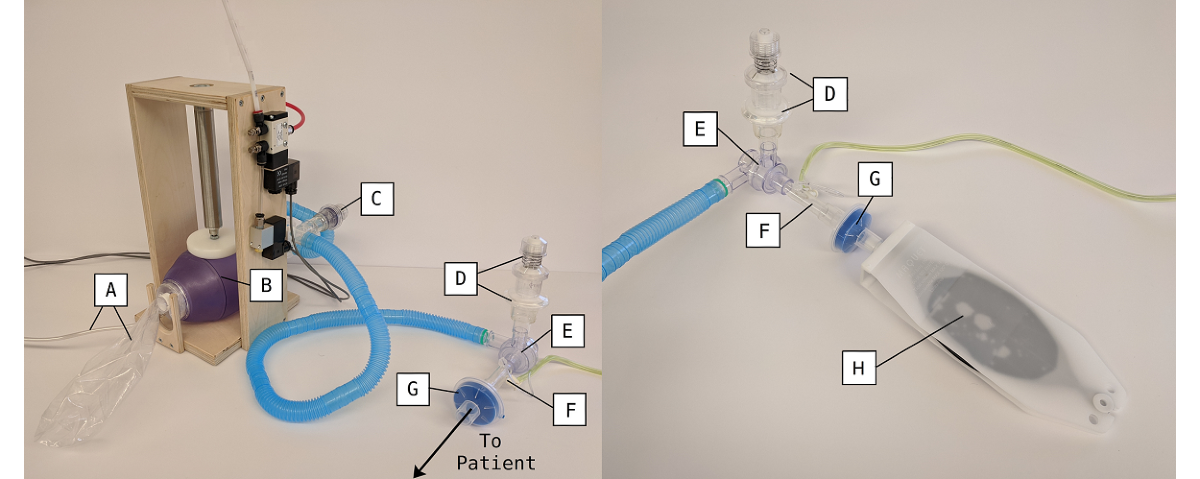
Patient circuit (left) and patient connection to test lung (right): (A) patient air supply hose and reservoir bag; (B) self-inflating bag; (C) peak inspiration pressure valve; (D) HEPA (high-efficiency particulate absorbing) filter and positive end-expiratory pressure valve; (E) active patient valve; (F) spirometer; (G) heat and moisture exchanger and filter; and (H) simple test lung.

The patient circuit (Figure 2) can be assembled from standard disposable hoses and components routinely used in hospitals, with 1 modification. The PIP valve is constructed from a PEEP valve by inserting a spring-compressing spacer into the valve. This results in a PIP valve with a pressure range of approximately 20 to 40 cmH_2_O (1 cmH_2_O is equivalent to 98.07 Pa).

#### Modes of Operation

**Figure 3:**
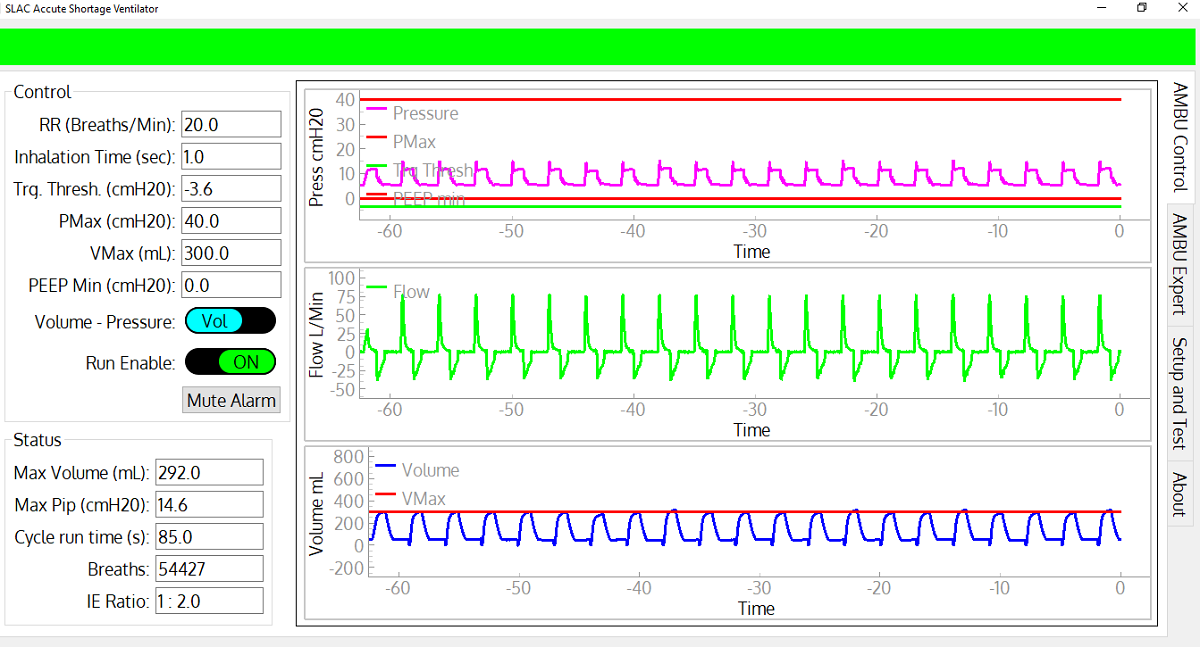
Screenshot of the control and monitoring graphical user interface. All primary controllable parameters are on the upper left and the time histories are displayed on the right. The major status values are on the lower left. The green banner on the top indicates no alarm conditions. Given an alarm, it changes to red or amber, flashes, and displays the alarm condition text.

The ASV has a graphical user interface (GUI) (Figure 3). The ASV can operate in volume-controlled assist control (VC-AC) mode or pressure-controlled assist control (PC-AC) mode. In both modes, the respiration rate and the inspiratory time can be set (or the inspiratory pressure that will trigger an assisted breath).

In VC-AC mode, a maximum volume is set. The flow measured by the spirometer is integrated to calculate the volume, and the bag compression is stopped when the maximum volume is reached. A proportional algorithm using a fraction of the difference between the set and actual maximum volume yields rapid convergence to the setpoint. In VC-AC mode, the PIP valve and maximum pressure setting of the microcontroller independently limit airway pressure.

In PC-AC mode, the target inspiratory pressure is set manually (Table 3) on the PIP valve. The pressure sensor provides an independent check on the pressure; it halts the bag compression for the current cycle if the maximum pressure parameter is exceeded, and it generates a high-priority alarm.

In both modes, the bag is held in its compressed state until the inspiratory time is reached. The bag is released, lowering the pressure, and exhalation occurs through the exhaust port of the patient valve into an optional PEEP valve (set manually). The cycle then repeats, triggered either by the respiration rate clock or by a voluntary inhalation.

**Table 3:** Controllable parameters.

Parameter name	Input	Description
Run enable	On/off	Controls the basic state of the ASV^a^
Control mode	Pressure/volume	Determines whether the ASV is limited by pressure or tidal volume
Respiration rate	Breaths per minute, in increments of 1 breath per minute	This parameter sets the minimal cycle rate for inhalation. Inhalations can occur more frequently by voluntary inhalation when pressure goes below the trigger threshold parameter. It is set from the GUI^b^ or control panel.
Inspiratory time	In seconds, in increments of 0.1 seconds	This parameter sets the time for the self-inflating bag compression and hold. It is set from the GUI or control panel.
PIP^c^ (maximum pressure)	In centimeters of water	This parameter is set by manually adjusting the PIP valve and by setting the maximum pressure parameter in the GUI or control panel. The PIP setting in the software provides an independent alarm if the PIP valve malfunctions.
PEEP^d^	In centimeters of water	This parameter is set by manually adjusting the PEEP Valve in the patient circuit.
PEEP, minimum	In centimeters of water	This parameter sets a warning threshold if the patient pressure goes below this value.
Trigger threshold	In centimeters of water	If the patient pressure goes below this threshold, an inspiration cycle is initiated. This threshold is compared against an absolute pressure gauge measurement, and is not relative to PEEP.
Maximum volume	In milliliters	This parameter sets the tidal volume limit by stopping the compression of the bag. An adaptive algorithm adjusts the internal microprocessor set-point based on the flow measurement to achieve reasonably precise volume control. This limit is always active, and does not require a different mode of ASV operation.

^a^ASV: acute-shortage ventilator.

^b^GUI: graphical user interface.

^c^PIP: peak inspiratory pressure.

^d^PEEP: positive end-expiratory pressure.

#### Monitoring and Alarm System

The ASV monitors status of electrical power and key operational data. It generates medium and high priority alarms annunciated by a sound generator and visual signals on the GUI and local display. The visual indications remain on the display until the alarm condition (Table 4) is no longer met.

**Table 4:** Alarm conditions.

Parameter	Priority level	Description
Electrical power lost	High	The 12 V electrical power is lost. The ASV^a^ cannot run in this state, but the alarms will be powered by the standby battery.
Maximum pressure	High	The patient-circuit pressure has exceeded the maximum pressure parameter. This indicates there may be a problem with the PIP valve, the hoses, the endotracheal tube, or the patient. The ASV continues running, but stops the compression of the bag at maximum pressure on each cycle.
Pressure low	High	The patient-circuit pressure is low. Possible reasons are the ASV has stopped due to loss of the pneumatic system or a disconnected patient hose.
Volume low	High or medium	In PC-AC^b^ mode, this is a high alarm when the volume drops below 250 mL. In VC-AC^c^ mode this is a medium alarm when the volume drops below 80% of maximum volume. The expected volume has not been met, possibly due to disconnected or kinked hose.
Volume high	Medium	In VC-AC mode, this is a medium alarm when the pressure exceeds 120% of maximum volume.
9 V battery low	Medium	The 9 V standby battery must be replaced to prevent failure of the Electrical Power Lost high-priority alarm. However, in the absence of this 9 V battery’s power, the ASV continues running normally.

^a^ASV: acute-shortage ventilator.

^b^PC-AC: pressure-controlled assist control.

^c^VC-AC: volume-controlled assist control.

#### Mechanical and Pneumatic Subsystems

A self-inflating resuscitator bag is compressed by a pneumatic cylinder inside a simple frame (Figure 1). The frame is made from 18 mm Baltic Birch plywood. The bag is loosely restrained by 2 plywood stanchions, and is easily replaced for a new patient. The pneumatic cylinder uses a 1-1/16-inch-diameter (27 mm) cylinder with a 4-inch stroke (100 mm). A 90-mm-diameter disk is attached to the end of the cylinder, which compresses the bag. The bag appears to be resilient enough for 1 month of steady operation.

The cylinder is controlled by a 3-port 2-position pneumatic valve which connects the cylinder to the air supply or to an exhaust control valve. This exhaust valve (PV2) holds air in the cylinder, which allows the piston to be held down until the inspiratory time has been reached.

The ventilator requires a source of compressed air (approximately 350 kPa). This is usually available from a central source in hospitals but also could be provided by cylinders of compressed air or by air compressors. The ASV consumes approximately 3 L per minute at a respiration rate of 20 brpm, thus a standard K-size gas cylinder would support a ventilator for approximately 30 hours. This is likely approximately half the rate of oxygen consumption.

**Figure 4:**
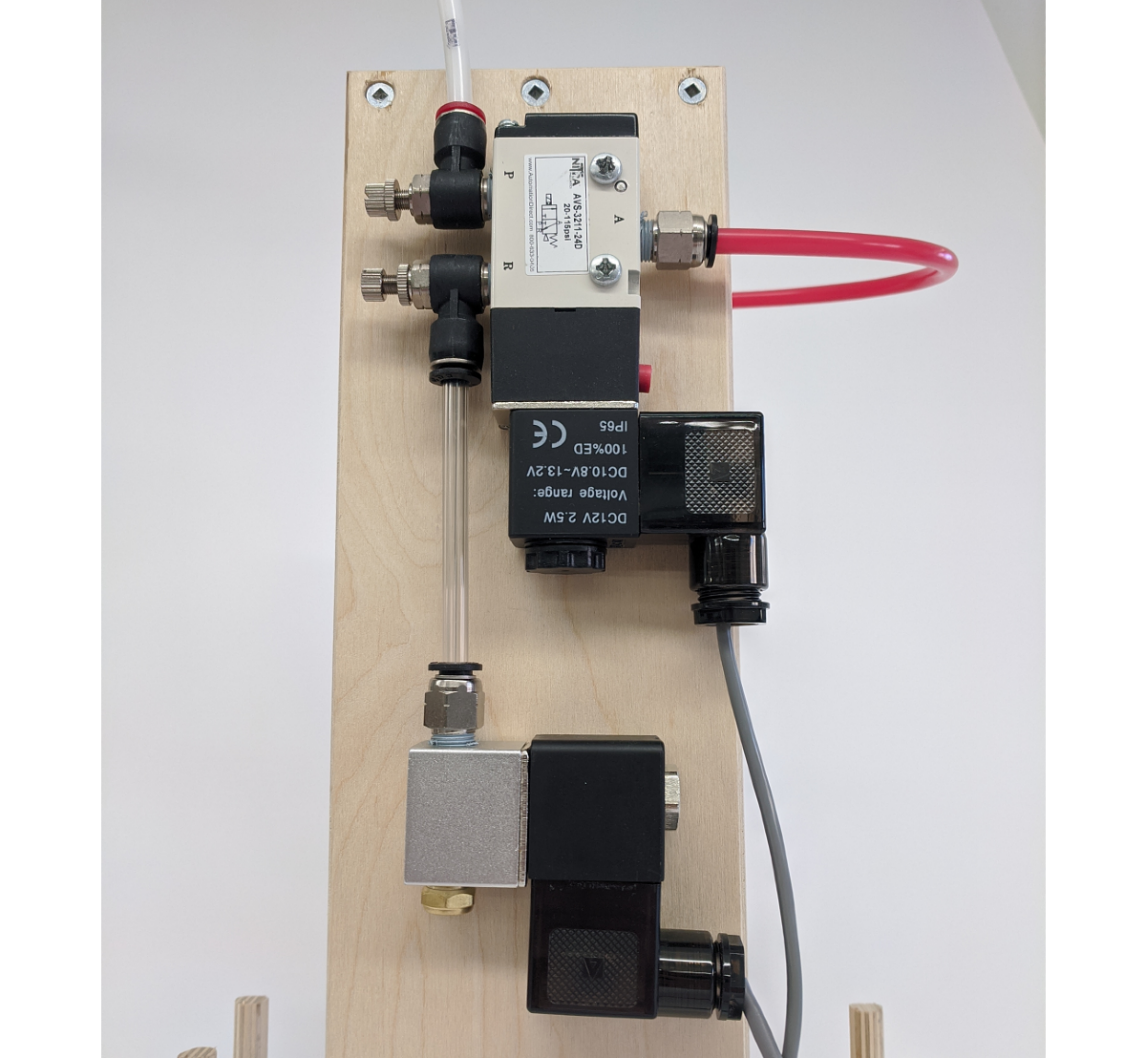
Pneumatic valves. The compressed air input is in the upper left.

The pneumatic circuit is shown in Figure 4. Two manual adjustable flow valves are used to control the piston compression and return rates. The 3-port 2-position pneumatic valve controls the piston down-stroke and dwell, and exhaust valve controls the return of the piston. This design allows the piston to stop when the desired volume is reached and maintain pressure in the self-inflating bag for the inspiratory time.

#### Sensors

There are 2 pressure sensors used to determine the flow and pressure of air in the patient circuit. The sensors have a sample rate of 100 Hz. The flow measurement is performed by a differential pressure sensor (Superior sensor SP110SM02, 211-SP110-SM02-R, Mouser) with an adjustable range, set to 5 cmH_2_O. Flow is calculated from the pressure drop measured across the spirometer. The patient airway pressure is also measured using a differential pressure sensor (Amphenol pressure sensor, DLC-L20D-D4, Amphenol All Sensors Corporation), with 1 of the 2 ports connected to the spirometer and the other open to atmospheric pressure. This sensor has a range of 50 cmH_2_O.

**Figure 5:**
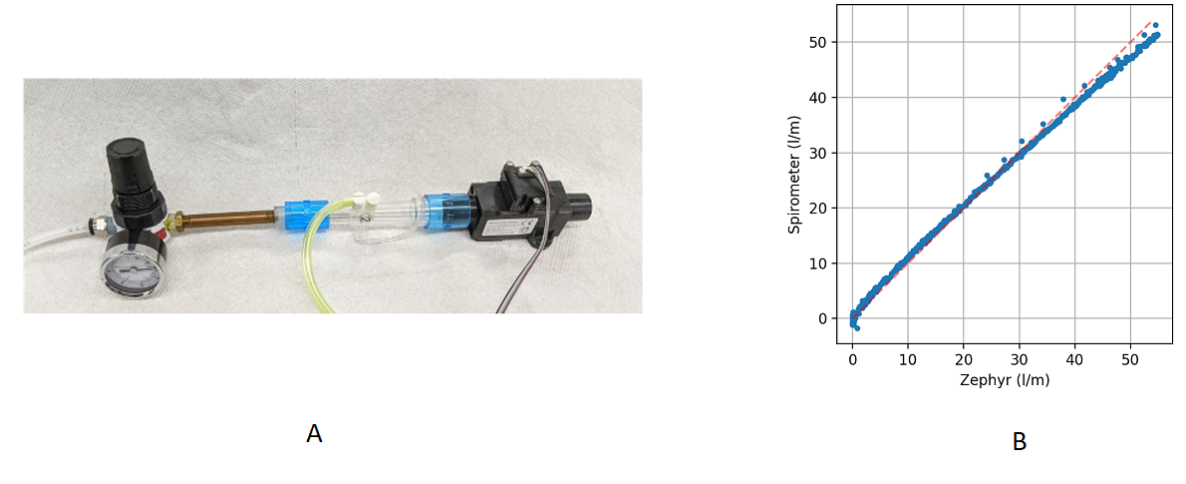
(A) Calibration setup, showing the spirometer in series with the flow meter (Zephyr) and a small pressure regulator to sweep the flow and (B) calibration results.

The spirometer was initially calibrated with a high-accuracy flowmeter (Zephyr HAFUHH0050L-4A-X, Honeywell) (Figure 5). The spirometer was placed in series with the flowmeter, and a low-pressure regulator was used to sweep the flow rate through the 2 instruments. The spirometer flow is proportional to the square root of the differential pressure, and the coefficient was determined from the measurements.

#### Electronics

A primary microcontroller (Nano 33 IoT, Arduino) controls the operation of the ASV. A second microcontroller (Nano 33 IoT, Arduino) runs the integrated LCD display and input knob used to display data and parameters and for setting ASV parameters when operating the device without a computer. Two microcontrollers allow the separation of critical real-time control of the ASV from the noncritical display and human input. Both microcontrollers are on a custom circuit board that provides connectors for power input (12 V DC), battery backup (9 V), computer data output (RS232 connection), and connectors to the external pressure sensor box and solenoid valves. Main power is provided to the ASV by a separate medical grade 36-watt AC-to-DC power converter, which avoids introducing line power into the enclosure. Internal backup power is provided via a single 9 V battery, which serves only to maintain the LCD display and alarm capabilities. An external UPS is required for fully functional uninterruptible power backup to the ASV.

#### Software

The software consists of 3 separate components: real-time control, display control, and a full GUI.

A real-time control component implemented in C++ runs on the primary microcontroller. It reads the sensors, operates the valves based on sensor data, time and configuration settings, and sends sensor data via separate serial interfaces to the display controller and, if connected, to the laptop computer. It can also receive configuration commands through the serial connections. Since it is the most critical software component for operation, it has been kept simple for easy review and great care has been taken to make sure communication with the other components cannot block its operation.

A display control component, also implemented in C++ runs on the secondary display control microcontroller. It receives and displays real-time information, manages a simple configuration menu controlled by an encoder knob, and sends configuration parameter updates to the real time controller.

A full GUI (Figure 3), implemented in Python (version 3), runs on a laptop. It is not required for normal operation but when connected provides the most comprehensive graphical display of system status, sensor data and calculated values. Unlike the built-in display, the GUI provides access to all configuration parameters. The GUI software has been designed to handle disconnects-reconnects gracefully and to clear its state when it detects a new ASV so that a single laptop can be used to control multiple ASVs by connecting to them one at a time.

The embedded software should be preloaded on the microcontrollers. The GUI software for the external laptop should be distributed on memory sticks or equivalent with the system and requires an existing Windows 10 (Microsoft Inc) installation. All software components can be upgraded in the field if necessary.

### Operation of the ASV

#### General

A laptop loaded with the GUI executable should be used to verify the functionality of each ASV unit before use. After connection of the services described above and connecting the laptop to the ASV, the GUI should be active. The user is guided through a setup and checkout procedure.

#### Setup and Test

The Setup and Test tab uses a sequence of scripts and commands to the primary controller to guide the user through this phase, with the ASV and patient circuit connected to a simple test lung. The protocol ensures that the flow and pressure sensors are properly connected, the system is reasonably leak tight, and the PIP Valve vents at its indicated top pressure. Both the VC-AC and PC-AC modes are tested for normal functionality, and the alarm functionality is tested by exceeding a set maximum pressure in PC-AC mode.

#### Control Interface

The pressure flow sensors are used for time history displays, along with a volume computed by integrating the flow. The respiration rate and the inspiratory time are settable. If the patient is not breathing voluntarily, the ASV will operate with the set respiration rate. If the patient begins to inhale before the next scheduled inhalation, which is indicated by the patient circuit pressure dropping below the operator-specified trigger threshold, a ventilator inhalation cycle will be initiated and the timing cycle is reset.

### Performance Testing

#### Overview

The ASV was subjected to performance testing during development and to verify the performance of the final device. Early testing was performed using simple rubber-bladder test lungs; later testing was performed using a Michigan Test Lung (2601i Michigan Instruments, Inc). The Michigan lung allowed for adjustable compliance and resistance testing. Two rounds of comprehensive tests were also performed in a hospital simulation center to determine if the ASV functioned correctly and achieved system requirements. A total of 29 scenarios, each with their own set of patient and ventilator variables, were created in collaboration with health care experts. The simulated scenarios addressed both healthy and unhealthy lungs, with both passive and active breathing to test the ASV in realistic situations.

#### Test Setup of a High-Fidelity Active Servo-Lung Simulator

Final testing was conducted testing using a high-fidelity lung simulator (Active Servo Lung, ASL-5000, IngMar Medical Ltd) at the Veterans Affairs Palo Alto Health Care System’s Simulation Center. The ASL is servo-controlled such that it can replicate, with high precision, a number of parameter settings for different aspects of patient lung mechanics. The models include lung compliance, inspiratory and expiratory resistance, and complex multiparameter models of the pattern of spontaneous breathing.

Tests with a simulated completely passive patient (ie, the equivalent of deep sedation and medical drug-induced neuromuscular paralysis) and with simulated spontaneous patient breathing were performed. Settings for spontaneous breathing used a half-sinusoidal profile of inspiration with the given patient respiratory rate (breaths per minute), maximum inspiratory pressure (−5 or −2 cmH_2_O), inspiratory time (20% of cycle), and expiratory release time (15% of cycle). We did not perform tests with a patient inspiratory pause or patient active expiration. Maximum inspiratory pressure is the largest negative pressure generated by the patient’s own breathing. Since ventilators are commonly set to trigger a mechanical breath at a patient breath of between −2 and −5 cmH_2_O, we used these settings to test the ability of the ASV to trigger correctly.

The ASL-5000 was equipped with a thorax, head, and neck manikin which allowed the insertion of an endotracheal tube (internal diameter: 7.0-8.0 mm) to replicate the scenario and tube resistance of a patient requiring mechanical support or ventilation.

#### Test Scenarios

Each test used a specific lung model and inspiratory pattern and lasted at least 20 steady-state breaths. This condition was reached quickly for the passive simulated patient but took longer for the simulated patient was breathing spontaneously. Data were sampled at 64 Hz. The variables used in this analysis were flow, airway pressure, lung pressure, muscle pressure, and volume. For each breath, the following parameters (derived values) were determined: minimum pressure (typically, the set PEEP), maximum pressure (PIP), respiratory rate (brpm), the ratio of inspiratory to expiratory time, and the integral of the flow (tidal volume). For every derived parameter, we report the mean and standard deviation.

Multiple waveforms were visible in real-time for inspection, and the waveforms and computed results were stored automatically by the simulator.

Tests covered different lung and breathing models (Table 5). The acute respiratory distress syndrome tests were performed with 3 different severities by setting the lung compliance and resistance. Spontaneous ventilation varied for respiration rate (10-30 brpm) and maximum inspiratory pressure (−2 or −5 cmH_2_O). For each lung model, several tests were performed with different ASV settings (Table 6). The selected ranges were based on AAMI CR503:2020 [10] design specifications and medical expertise.

**Table 5:** ASL-5000 settings for different lung models.

Model	Lung compliance (mL/cmH_2_O)	Inspiratory resistance (mL per s cmH_2_O)	Expiratory resistance (mL per s cmH_2_O)
Normal	50	6	6
Acute respiratory distress syndrome 1	30	6	6
Acute respiratory distress syndrome 2	30	11	16
Acute respiratory distress syndrome 3	10	20	20

**Table 6:** Ventilator settings for volume- and pressure-control ventilator modes.

	Respiratory rate (brpm)	Set tidal volume (mL)	Peak inspiratory pressure (cmH_2_O^a^)	Inspiration time (seconds)	Inspiratory-to-expiratory ratio	Trigger threshold (cmH_2_O)	Positive end-expiratory pressure
Volume-controlled assist control	10-30	250-600	15-35	0.6-1.5	1:1 to 1:4	–2 to –5	5-20
Pressure-controlled assist control	20-40	N/A^b^	25-35	1.0	1:1 to 1:2	N/A	5-20

^a^1 cmH_2_O is equivalent to 98.07 Pa.

^b^N/A: not applicable.

To demonstrate that the ASV achieves the tidal volume range requirements (Table 1), we tested the ventilator in both VC-AC (volume-controlled assist control) and PC-AC (pressure-controlled assist control) modes. In PC-AC mode, these measurements show the maximum tidal volume that the ASV can deliver. For the healthy lung, an additional measurement was made at a lower PIP. For the acute respiratory distress syndrome configurations, the PEEP Valve was manually set to 10 cmH_2_O. PEEP values of 10, 15, or 20 cmH_2_O are consistent with what is commonly utilized for patients with significant acute respiratory distress syndrome [10]. The respiratory rate was 20 brpm, the inspiratory-to-expiratory ratio was 1:2, and the PIP valve was set to 35 cmH_2_O. The ASV was tested on a healthy lung in VC-AC mode with respiratory rates between 10 and 30 brpm; only the inspiratory time was changed to change the inspiratory-to-expiratory ratio. Measurements of the inspiratory to expiratory time for different inspiratory times were acquired with a set tidal volume of 400 mL, a PEEP value of 5 cmH_2_O, and a PIP value of 35 cmH_2_O.

The ability of the ASV to provide spontaneous breaths, in compliance with AAMI CR503:2020 [10], was demonstrated using the ASL-5000 in active mode (30 brpm with an inspiratory pressure of −5 cmH_2_O) coupled to the ASV (in VC-AC mode; 20 brpm; tidal volume: 400 mL, PEEP 5 cmH_2_O; trigger threshold 3 cmH_2_O; ASV trigger threshold pressure with respect to PEEP: −2 cmH_2_O).

Breathing through a disabled ASV was tested by an author breathing through a mouthpiece connected to the spirometer port. The ventilator was set to only record data.

## Results

### General

**Figure 6:**
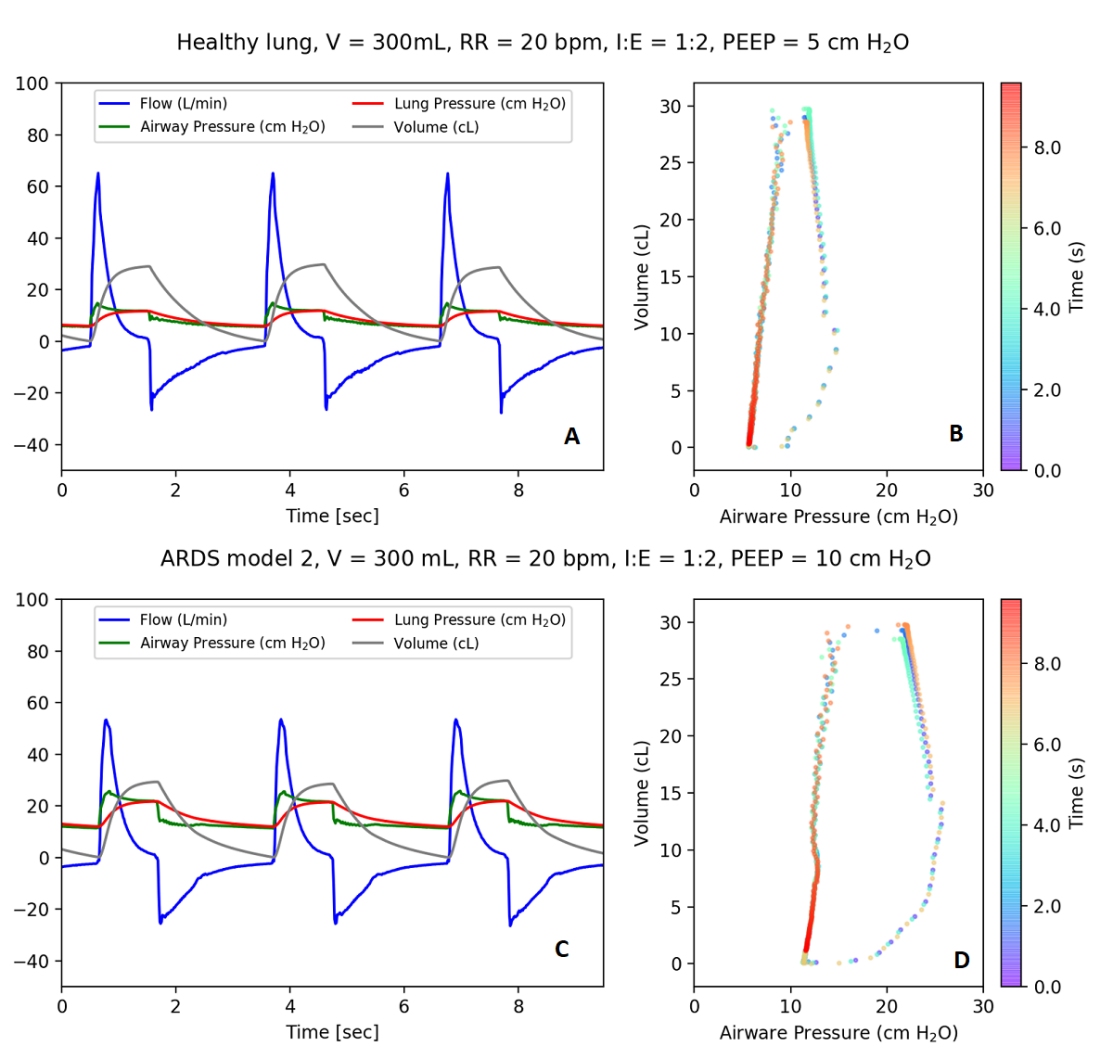
Example waveforms. (A) Ventilator connected to the simulator and (B) pressure–volume curve over time for healthy lung model and (C) ventilator connected to the simulator and (D) pressure–volume curve over time for with an acute respiratory distress syndrome 2 lung model. In A and C, flow is shown in blue, pressures are shown in red and green, and volume is shown in gray. ARDS: acute respiratory distress syndrome; I:E: inspiratory-to-expiratory ratio; PEEP: positive end-expiratory pressure; RR: respiratory rate; V: volume.

In Figure 6, example steady-state breaths are shown when the same ventilator settings (in VC-AC mode) are used for 2 different lung models (except PEEP, which acts as a pressure offset). A single breath starts with flow rising rapidly, which is caused by the piston moving down and squeezing the self-inflating bag. The rate that the piston descends is adjustable by the needle valve input to the pneumatic system, which is set to approximately half a second, until it hits the maximum volume setpoint and is held in place. During this time, the pressure remains high but the flow stops—what the patient would experience as an inspiratory pause—which increases the time oxygen can be absorbed in the lungs.

Once the inspiratory time ends, the piston moves upwards and the simulated patient exhales. The start of exhalation is shown by the decreasing volume, after it has reached its maximum, and by the flow reversal. The difference between measured lung and airway pressure is due to the resistance of the lungs in the simulated patient. The acute respiratory distress syndrome models require a higher pressure for the same volume of air in comparison to a healthy lung; the ASV delivers a set tidal volume of 300 mL in 2 different lung scenarios by utilizing increased pressure when required for a patient with acute respiratory distress syndrome.

### Demonstration of Performance Requirements

**Figure 7:**
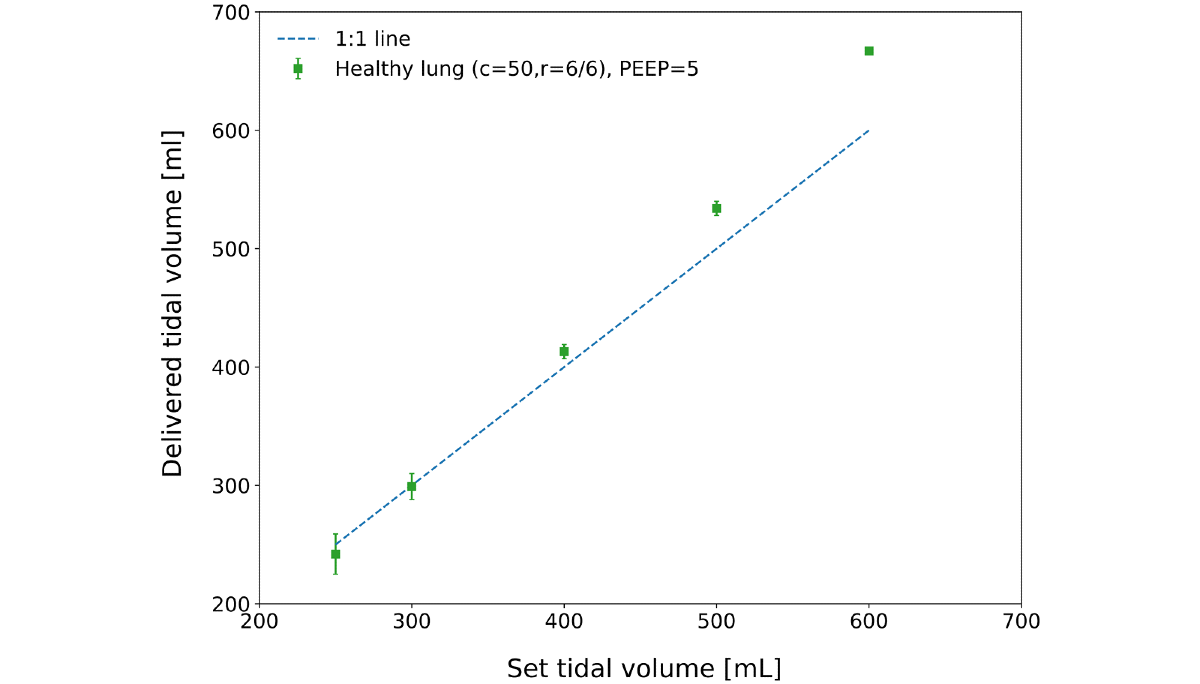
Healthy lung model tidal volumes (delivered vs set) in volume-controlled assist control mode connected to a passive lung. The dashed line is show departures from one-to-one linearity. c: compliance; PEEP: positive end-expiratory pressure; r: resistance.

Figure 7 shows the delivered tidal volume versus the set tidal volume for the ventilator in VC-AC mode while connected to the ALS-5000 normal lung model. For 5 measurements, over a total range of 250 to 600 mL, the delivered tidal volume was within the 15% required tolerance.

**Figure 8:**
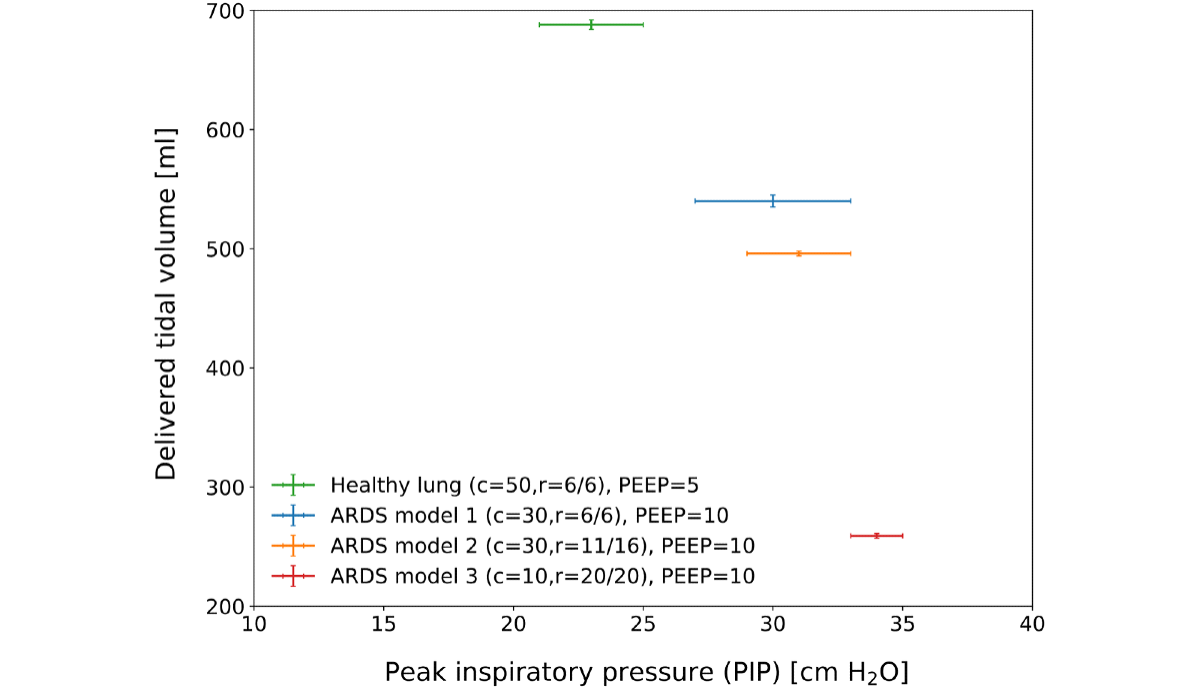
Delivered tidal volume as a function of set peak inspiratory pressure with the ventilator in pressure-control mode connected to the ASL–5000 set to passive-patient mode. ARDS: acute respiratory distress syndrome; c: compliance; PEEP: positive end-expiratory pressure; r: resistance.

Figure 8 shows the delivered tidal volume and measured PIP in PC-AC mode for 5 different ventilator settings. For the healthy lung, the measurement was made at a lower PIP, resulting in a lower delivered tidal volume. A higher PIP was required to reach the maximum tidal volume for more severe acute respiratory distress syndrome models. These results show that the ASV in VC-AC or PC-AC modes achieves the required tidal volume range for all scenarios, except for the most extreme acute respiratory distress syndrome 3 model, where it achieved a tidal volume of only 259 mL.

It achieved the required respiratory rate range of 10 to 30 brpm within the tolerance of 2 brpm.

For a respiratory rate of 20 brpm, the breath cycle time was a total of 3 seconds. The piston speed was set with the needle valve to achieve a full downward stroke in 0.5 seconds; the rest of the inspiratory time is thus used as an inspiratory pause. During the inspiratory pause, the pressure is kept constant so that the lungs stay fully open before the piston retracts and the patient exhales. The ASV was able to achieve the required range of the inspiratory-to-expiratory ratio of 1:1 to 1:4 (Table 7). PIP values for all 29 tested scenarios stayed below the required 40 cmH_2_O. The highest reported PIP value was 34 cmH_2_O (SD 2).

**Table 7:** Set and measured inspiratory-to-expiratory time for the ASV in VC-AC mode connected to a passive lung.

Set values				Measured value
Respiration rate (brpm)	Inspiratory time (seconds^a^)	Inspiratory-to-expiratory ratio^b^	Inspiratory-to-expiratory ratio	
20	1.5	1:1	1:0.9	
20	1	1:2	1:2.0	
20	0.75	1:3	1:2.9	
20	0.6	1:4	1:3.9	

^a^The measured uncertainty on the inspiratory-to-expiratory ratio is less than the rounding error.

^b^The required range of the inspiratory-to-expiratory ratio is 1:1 to 1:4 [10].

**Figure 9:**
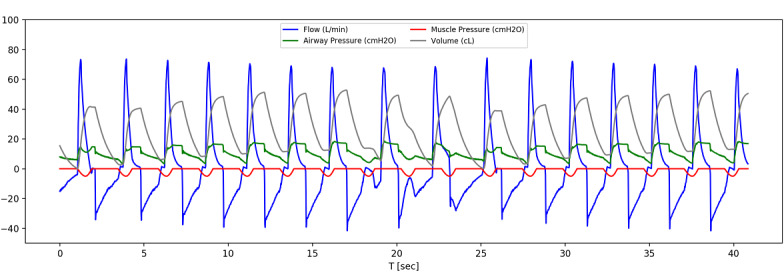
Example waveforms when the acute-shortage ventilator (ASV, set to 20 brpm) is triggered by spontaneous breathing of the lung simulator (30 brpm). The muscle pressure (red line) shows when the spontaneous breaths are requested by the lung simulator, while the start of the positively increasing flow (blue line) shows when the ASV delivers a breath.

The ASV provided spontaneous breaths, in compliance with AAMI CR503:2020 [10]. A spontaneous simulator breath is started by decreasing the inspiratory muscle pressure (Figure 9). When the airway pressure drops below the trigger threshold the ASV starts a breath, indicated by a sharp (positive) rise in the flow. If the ASV respiration rate is small compared to the spontaneous breathing rate then the breathing cycles can be missed because the airway pressure does not fall below trigger threshold (evident at approximately 17 seconds, Figure 9). If the trigger threshold is not met in the time window set by respiration rate, a breath will be provided by the ASV at the appropriate time. The ASV is correctly triggered for most breaths, but because the exhalation time setting is too short at 30 brpm, the pressure does not reliably fall below the trigger threshold before the simulated patient requests the next breath. With these settings, approximately 1 in 10 spontaneous breaths is missed (reverts to the ventilator timing).

**Figure 10:**
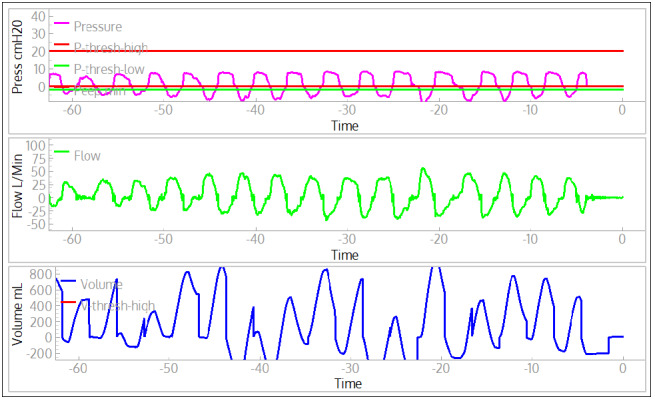
Example waveform of spontaneous breathing (one of the authors) with ventilator in monitoring mode.

Breathing was possible through a disabled ASV (Figure 10) because there is an inlet valve on the self-inflating bag. In any case, this causes a high priority alarm.

## Discussion

In this paper, we described the design, implementation, and testing of a low-cost easy-to-manufacture ASV for potential use by medical professionals during shortages of standard equipment, which was motivated by the COVID-19 pandemic. Our device consists of a patient circuit based primarily on a readily available self-inflating resuscitator bag and other standard intensive care unit parts, with a pneumatic plunger system to automatically compress the bag based on adjustable parameters. The patient circuit consists of only standard hospital parts to help medical professionals have sufficient confidence in the device should they require its use for treating patients in their communities. Additionally, by selecting primarily nonmedical components for the compression system, as well as related sensors and control elements, we sought to have a design that took advantage of items in industrial supply chains as much as possible. Pressure and flow are measured by modern differential pressure sensors that are read by microcontrollers and permit volume- or pressure-controlled assisted breathing. The ASV can be operated standalone with the integrated display and controls. An optional computer and GUI provide full time-history plots.

The development of the ASV was carried out through a partnership of SLAC National Accelerator Laboratory scientists and technical staff, and medical professionals from 3 Bay Area medical centers, the Stanford University School of Medicine, Santa Clara Valley Medical Center, and the Veterans Affairs Palo Alto Health Care System. Technical development and testing began with simple rubber-bladder test lungs, was followed by design feedback based on tests with a Michigan Test Lung, and 2 rounds of performance tests were performed with the ASL-5000. The final round of tests with the demonstrated that the ASV meets all requirements defined by AAMI CR503:2020 [10]. The combination of high-performance, easy manufacturability, and low-cost of the ASV may make it an effective tool in combatting the COVID-19 pandemic; however, the ASV has not yet been submitted to the US Food and Drug Administration for certification.

## Abbreviations

AAMI: Association for the Advancement of Medical Instrumentation
ASV: acute-shortage ventilator
GUI: graphical user interface
IEC: International Electrotechnical Commission
ISO: International Standards Organization
PC-AC: pressure-controlled assist control
PEEP: positive end-expiratory pressure
PIP: peak inspiration
VC-AC: volume-controlled assist control
